# A retrospective analysis of characteristics of visual field damage in patients with Leber’s hereditary optic neuropathy

**DOI:** 10.1186/s40064-016-2540-7

**Published:** 2016-06-23

**Authors:** Ruijin Ran, Shuo Yang, Heng He, Shiqi Ma, Zhiqi Chen, Bin Li

**Affiliations:** Department of Ophthalmology, Tongji Hospital, Tongji Medical College, Huazhong University of Science and Technology, 1095 Jie-fang Road, Wuhan, 430030 Hubei China; University Hospital of Hubei University for Nationalities, Enshi, 445000 Hubei China

**Keywords:** Leber’s hereditary optic neuropathy, Retrospective analysis, Visual field

## Abstract

The objective of this study is to investigate the characteristics and the evolution of visual field damage caused by Leber’s hereditary optic neuropathy (LHON) and to provide clinical data for the diagnosis of LHON. Parameters of visual field in 32 consecutive patients (49 eyes) with LHON who were confirmed by genetic diagnostic tests were retrospectively measured within 1 week, between three to six months, and at six months after onset. Visual field defects revealed central scotoma in 26 eyes (53.1 %), paracentral scotoma in 12 eyes (24.5 %), ceco-central defects in 6 eyes (12.2 %), blind spot enlargenment in 3 eyes (6.1 %), quadrantanopia in 2 eyes (4.1 %) within 1 week after onset. After 3 to 6 months, ceco-central defects were detected in 22 eyes (44.9 %), central isopter constriction in 10 eyes (20.4 %), hemianopia or quadrantanopia in 5 eyes (10.2 %), central scotoma in 4 eyes (8.2 %), and paracentral scotoma in 1 eye (2.0 %). After 6 months, central isopter constriction was observed in 18 eyes (36.7 %), diffuse defects in 21 eyes (42.9 %), ceco-central defects in 3 eyes (6.1 %), hemianopia or quadrantanopia in 5 eyes (10.2 %), and central scotoma in 2 eyes (4.1 %). LHON at different stages was characterized by different focal visual field defects: visual field defects in LHON patients within 1 week after onset were mostly central or paracentral scotoma, which was enlarged around the ceco-central defect, or connected to form a blind spot after 3–6 months. Diffuse and central isopter constriction defects were usually developed after 6 months. Damages firstly appeared in papillomacular bundle and gradually expanded outward. These characteristics of visual field defects reported in this study might provide a clinical basis for better diagnosis of LHON.

## Background

Leber’s hereditary optic neuropathy (LHON) has a mitochondrial inheritance pattern. In 91–92 % of cases in mainland and Taiwan of China, the disease is associated with G11778A mitochondrial DNA (mtDNA) mutations (Lin et al. 2012; Wang et al. 2005). LHON typically affects young adult males who have the gene mutation presenting unilateral or bilateral subacute/acute painless loss of central vision. The disease has usually entered middle or late phase when diagnosed (Hilo et al. 2013; Man et al. 2002; Vergani et al. 1995).

In the early stage of the disease, the unmyelinated retinal nerve fibre layer of optic nerve is firstly affected, especially the papillomacular bundle. With the development of the disease, the entire nerve fibre is affected and optic atrophy ensues. According to the evolution of LHON, the visual field defects is also typical. Firstly, there is a cecocentral defect, and then a larger central defect, often with a superior or inferior predilection (Wallace et al. 1988; Yu-Wai-Man et al. 2009).

We have previously conducted a few studies on the development of effective gene therapies for the treatment of LHON (Cui et al. 2013; Pei et al. 2013; Shi et al. 2012; Yang et al. 2015). In this retrospective study, visual field for 32 patients (49 eyes) with LHON who were confirmed by genetic diagnostic tests were measured within 1 week, between three to six months, and at six months after onset to investigate the characteristics of visual field damage caused by Leber’s hereditary optic neuropathy (LHON).

## Methods

### Subjects

A total of consecutive 32 patients (49 eyes) who were diagnosed as LHON by mtDNA analysis between January 2013 and July 2015 in the Department of Ophthalmology, Tongji Hospital, Tongji Medical College at the Huazhong University of Science and Technology, China were selected in this study. The study was a retrospective design and was approved by the ethics committee in Tongji Hospital at the Tongji Medical College, and performed strictly following the provisions of the Declaration of Helsinki. Visual field testing and others ophthalmologic examinations including slit-lamp, BCVA, intraocular pressure, and fundus examination of all patients were reviewed. Professor Ni evaluated the disease stage for each patient as previously described (Nikoskelainen et al. 1984). Disease were categorized into three stages: onset (within a week), middle stage (between 3 and 6 months), and late stage (over 6 months), the average time of the 3groups were 0.13 ± 0.06, 4.4 ± 0.88, 10.47 ± 3.4 m respectively. Eyes with intraocular pressure above 21 mmHg, retinal disease, unclear history, other optic neuropathies or no visual field damage were excluded. The study was a retrospective design and was approved by the ethics committee in Tongji Hospital at the Tongji Medical College, and performed strictly following the provisions of the Declaration of Helsinki.

### Visual field examination

Visual field examination was performed using HFAII740 (Humphrey Field AnalyzerII, Carl Zeiss Meditec, Dublin, California, USA) by an experienced operator in a darkroom.

Patients received the original pupil dilation and inhibited mydriasis with the following parameters: 1. the duration of stimulation cursor was 200 ms; 2. the brightness of background light was 31.5 ASB; 3. the brightness of stimulation cursor was 0.08–10,000 ASB; 4. the range of measurement was 0°–30° central field. A total of 5.76 points were measured. The following results were recorded: 1. fixation losses, 2. false NEG errors, 3. false POS errors, 4. mean defect (MD), 5. pattern standard deviation (PSD), 6. visual field index (VFI). Visual test showed that fixation loss more than 20 % and false positive or false negative errors greater than 15 % had been excluded. The PSD reflects the variation of light sensitivity and the local vision defects; the MD shows whether the light sensitivity of general visual field defects and how much it defects; the VFI tends to reflect the overall level of the central visual field. The Visual field defects were classified as paracentral scotoma, ceco-central defects, blind spot enlargenment and quadrantanopia. Cecocentral defects were defined as isolated scotomas in the circular area between 0° and 10°. Paracentral scotoma was defined as isolated scotomas in the Bjerrum’s area between 5° and 25°. BCVA examinations were performed using an international standard visual acuity chart. The category of the visual field of each patient was defined by another doctor Zhiqi Chen, who did not know the stage of disease for each patients.

### Statistical analysis

Data were presented as mean ± standard deviations. All data were analyzed using SPSS software (version13.0). Variations between groups were compared by post hoc tests. Correlations between VFI, MD and BCVA (Log MAR) were analyzed using Pearson correlation coefficient. Characteristics of visual field damage associated with LHON were analyzed. p values less than 0.05 were considered statistically significant.

## Results

A total of 32 consecutive LHON patients were selected for this study including 4 women and 28 men with a mean age of 22.8 (range: 9 to 48 years). All diagnosed patients were confirmed by mtDNA analysis including 29 cases (90.6 %) of G11778A, 2 cases of G3460A, and 1 case of T14484C.

### Sensitivity and stability of visual field

**Fig. 1:**
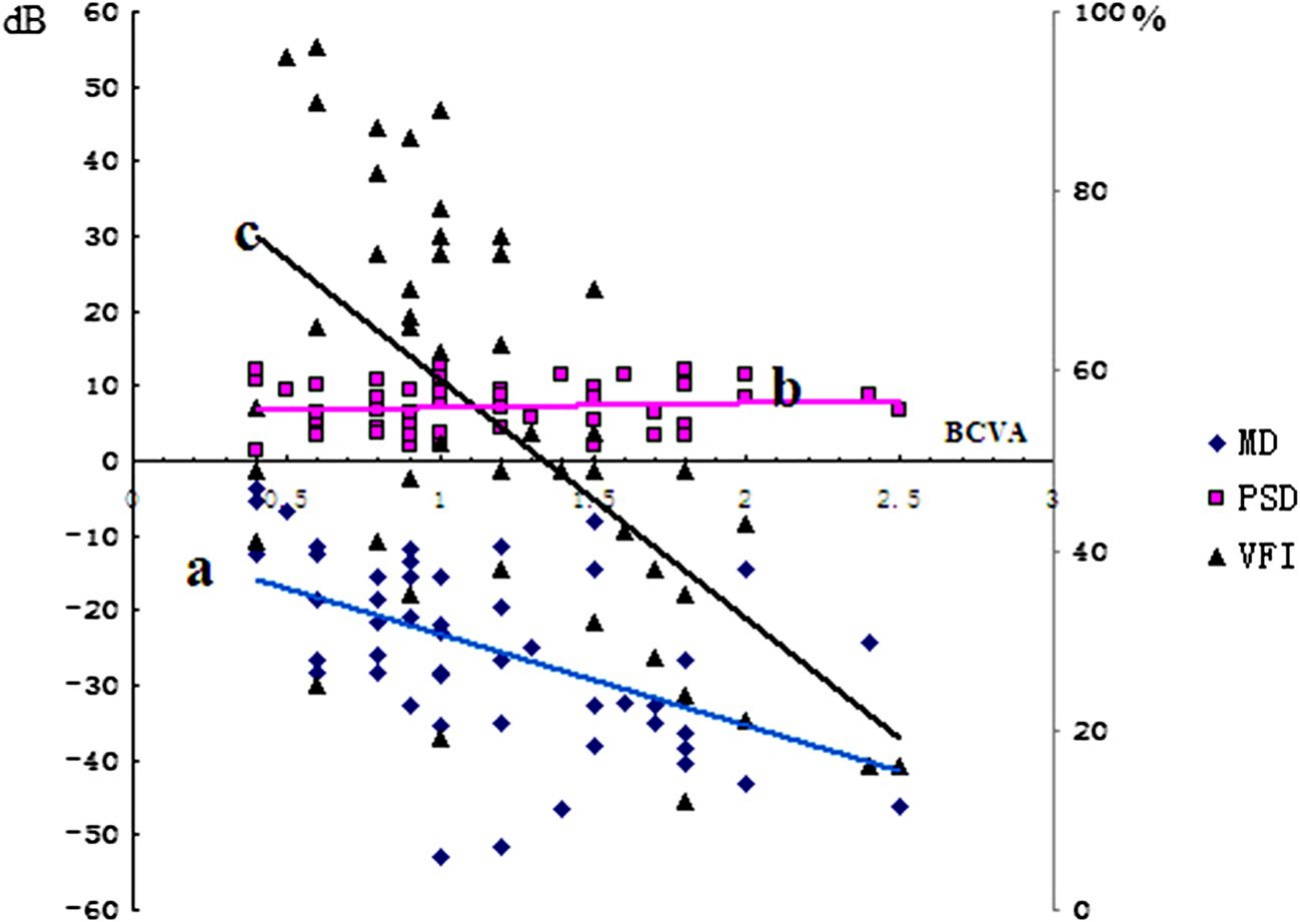
Correlations between VFI, MD and BCVA (LogMAR):MD was negatively correlated with BCVA (LogMAR) (r = –0.613, p = 0.011) (*line a*). VFI was negatively correlated with BCVA (LogMAR) (r = –0.703, p = 0.000) (*line c*). No correlation between PSD and BCVA (Log MAR) was detected (r = 0.102, p = 0.577) (*line b*)

Correlations between VFI, MD and BCVA (LogMAR) were analyzed using Pearson correlation coefficient, It was found that MD was negatively correlated with BCVA (LogMAR) (r = −0.613, p = 0.011). VFI was also negatively correlated with BCVA (LogMAR) (r = −0.703, p = 0.000). No correlation between PSD and BCVA (Log MAR) was detected (r = 0.102, p = 0.577) (Fig. 1). The visual field for 27 eyes with relatively stable BCVA (Log MAR) at 3 and 6 months was compared. It was shown that MD at 3 months was significantly lower when compared with that at 6 months (p = 0.029), whereas PSD (p = 0.619) and VFI (p = 0.125) at 3 months were similar to those at 6 months. The visual field of 22 eyes with unstable BCVA (LogMAR) was also analyzed. While MD (p = 0.028) and VFI (p = 0.016) at 3 months were significantly lower than those at 6 months, PSD at 3 months was similar to that at 6 months (p = 0.599). Although BCVA (LogMAR) has been considered as an important objective indicator for the evaluation of visual impairment in LHON patients, VFI and MD were confirmed as highly sensitive and stable indicators for LHON in our study.

### Characteristics of visual field damage

**Fig. 2:**
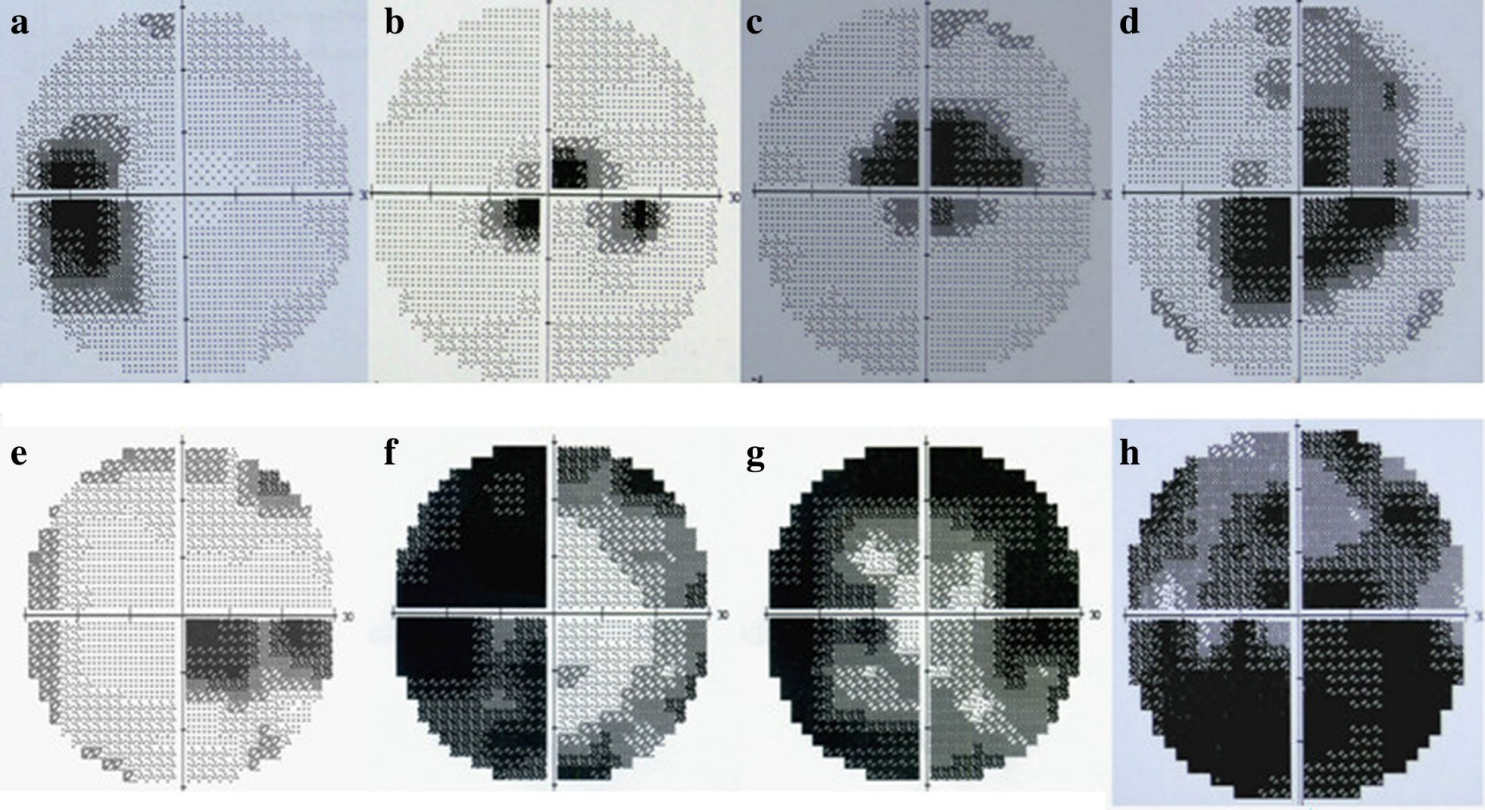
Visual field defects found in patients with LHON (from the first to the last): characteristics of visual field damage are blind spot enlargement (**a**), paracentral scotoma (**b**), central scotoma (**c**), ceco-central scotoma (**d**), hemianopia (**e**), quadrantanopia (**f**), central isopter constriction (**g**), diffuse defects (**h**)

**Fig. 3:**
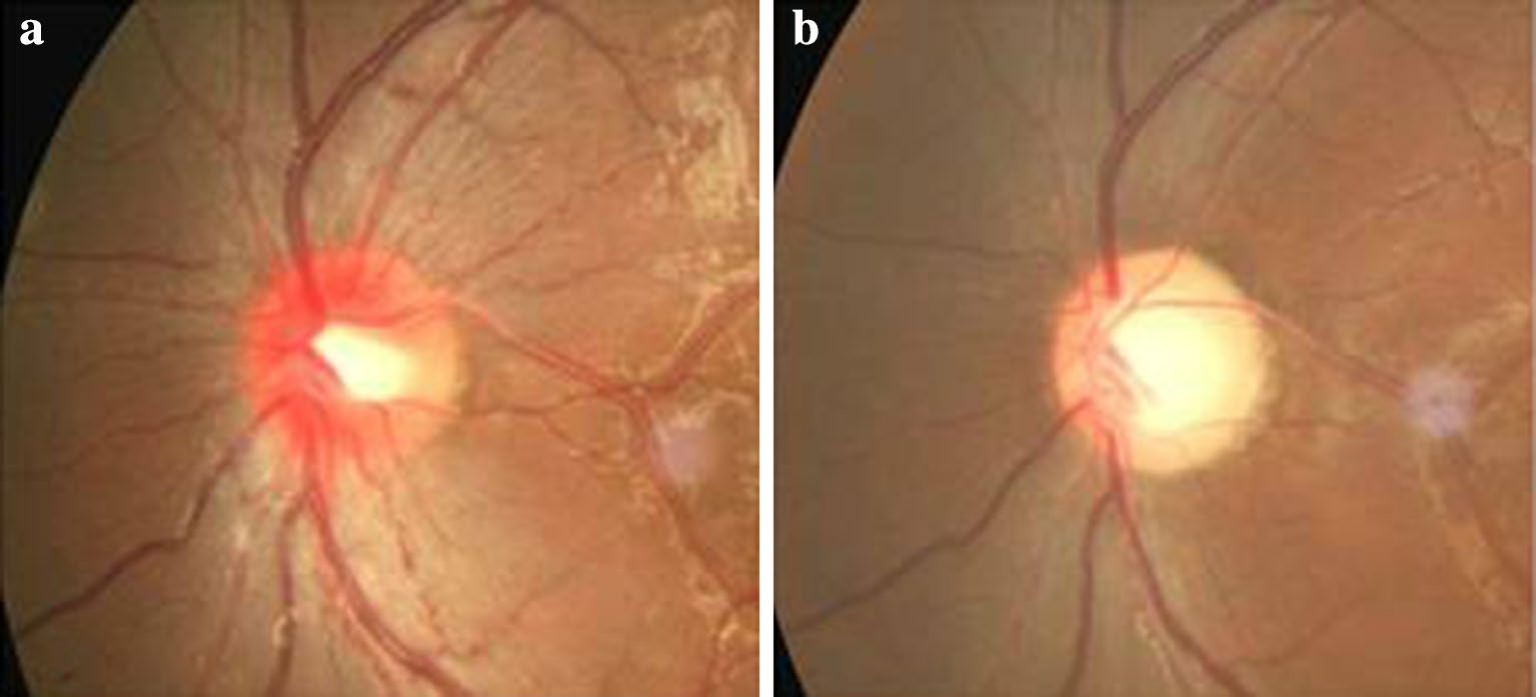
Characteristics of fundus in LHON patients. **a** The early manifestation of fundus (within 1 week): retinal vein and artery expanded around the optic disk. Edema and hyperemia surrounding the optic disc were observed. **b** The late stages (over 6 months): retinal vein and artery have become tapered and stiff. The optic disc is pale and atrophic

Visual fields were analyzed by doctor chen and professor liin 32 patients (49 eyes) with LHON. Central scotoma was revealed in 26 eyes (53.1 %), paracentral scotoma in 12 eyes (24.5 %), ceco-central defect in 6 eyes (12.2 %), blind spot enlargenment in 3 eyes (6.1 %), and quadrantanopia in 2 eyes (4.1 %) within 1 week after onset. From 3 to 6 months, ceco-central defect was detected in 22 eyes (44.9 %), central isopter constriction in 10 eyes (20.4 %), hemianopia or quadrantanopia in 5 eyes (10.2 %), central scotoma in 4 eyes (8.2 %), and paracentral scotoma in 1 eye (2.0 %). Six months later, central isopter constriction was observed in 18 eyes (36.7 %), diffuse defect in 21 eyes (42.9 %), hemianopia or quadrantanopia in 5 eyes ((10.2 %), ceco-central defect in 3 eyes (6.1 %), and central scotoma in 2 eyes (4.1 %). LHON at different stages was characterized by different focal visual field defects: visual field defects in LHON patients within 1 week after onset were mostly central or paracentral scotoma, which was enlarged around the ceco-central defect, or connected to form a blind spot after 3–6 months. Diffuse and central isopter constriction defects were usually developed after 6 months. Damages firstly appeared in papillomacular bundle and gradually expanded outward. Clinical characteristics of visual field defects during the progression of LHON are summarized in Table 1 and two illustrative cases are presented in Figs. 2 and 3. MD (dB), PSD (dB), VFI (%) mean ± SD and one-way ANOVA at different stages are summarized in Table 2.

**Table 1:** Characteristics of visual field damage associated with LHON

Type of visual field damage	These cases (percent) of visual field damage in different stages		
	Within 1 week	From 3 to 6 months	Over 6 months
Central scotoma	26 (53.1 %)	4 (8.2 %)	2 (4.1 %)
Paracentral scotoma	12 (24.5 %)	1 (2.0 %)	
Blind spot enlargenment	3 (6.1 %)		
Ceco-central scotoma	6 (12.2 %)	22 (44.9 %)	3 (6.1 %)
Diffuse defects		7 (14.3 %)	21 (42.9 %)
Central isopter constriction		10 (20.4 %)	18 (36.7 %)
Hemianopia or quadrantanopia	2 (4.1 %)	5 (10.2 %)	5 (10.2 %)

**Table 2:** MD (dB), PSD (dB), VFI (%) mean ± SD and one-way ANOVA at different stages

Index of visual field	Mean ± standard deviation and one-way ANOVA				
	Within 1 week	From 3 to 6 months	Over 6 months	F	p
MD	−25.2 ± 12.21	−30.6 ± 18.63	−36.3 ± 21.19	4.48	0.009
PSD	7.24 ± 3.25	9.67 ± 3.13	11.97 ± 2.87	28.62	0.000
VFI	75.69 ± 15.46	50.37 ± 21.53	36.45 ± 28.65	10.73	0.000

## Discussion

LHON was firstly reported by the VonGrafe in 1858. In 1871, the disease was named after Doctor Leber who discovered that LHON was an independent disease. In 1988, Wallace DC (Wallace et al. 1988) found that LHON was associated with mtDNA mutation. LHON typically appears in young males with clinical manifestation including central vision damage and acute or subacute, painless, binoculus or monocular vision loss (Mulliez et al. 2000). Numerous studies have reported the characteristics of visual field defects associated with idiopathic optic neuritis (ION). Marshall (1950) has detected no visual defects around central or paracentral scotoma in 90.8 % of ION cases. Trobe and Glaser (1978) have reported no hemianopia in 81 cases of ION, and considered hemianopia as an important indicator to differentiate ION from compressive optic neuropathy. In a study by Keltner et al. (1993), diffuse visual field defects are detected in 48.2 % of cases, whereas all other patients have developed focal visual field defects. Among these, the central scotoma only accounts for 3.8 %, including hemianopia and other types of visual field defects.

So far, the visual field defects due to LHON have been rarely reported and the characteristics of the visual field damage during the progression of the disease have been analyzed in a few papers (Newman et al. 2005, 2006), but these papers analyzed the visual field changes only several patients. Zhang et al. (2014) have detected a large central or paracentral scotoma at the early stage of LHON. La Morgia et al. (2014) have described the diversity of visual field defects associated with LHON, but failed to analyze the characteristics of these visual field defects with the progress of the disease. It has also been reported that the scotoma or its fenestration in some LHON patients is relieved (Nakamura and Yamamoto 2000). A component of the visual improvement in LHON may be the adaptive phenomenon of eccentric fixation (Altpeter et al. 2013).

In this study, we found that MD and VFI were negatively correlated with BCVA (LogMAR) in all tested eyes with or without stable BCVA (LogMAR), indicating the high sensitivity and stability of visual field examination in the diagnosis of LHON. We found that the visual field defects were mostly central (53.1 %) and paracentral (24.5 %) scotoma at early stages of LHON. As the disease progressed, the visual field defects exasperated around the scotoma, and extended from two sides or enlarged surrounding the blind spot. In the late phase, visual field defects mainly included central isopter constriction (36.7 %), diffuse defects (42.9 %), hemianopia or quadrantanopia (10.2 %), In contrast to early stage: visual field defects including the nasal step and arcuate scotoma such as early glaucoma damage were seldom detected at late stages of LHON. These characteristics of visual field defects reported in this study might be useful for differentiating the diagnosis of the disease from glaucoma.

In summary, LHON at different stages was characterized by different focal visual field defects: visual field defects in LHON patients within 1 week after onset were mostly central or paracentral scotoma, which was enlarged around the ceco-central defect, or connected to form a blind spot after 3–6 months. Diffuse and central isopter constriction defects were usually developed after 6 months. Damages firstly appeared in papillomacular bundle and gradually expanded outward. These characteristics of visual field defects reported in this study might provide a clinical basis for better diagnosis of LHON. Visual field examination might be applied to assess the improvement of visual function in LHON patients who have received gene therapy. However, since LHON is a rare disease, the current study has been limited by the small number of cases. Further studies with a much larger sample size are needed to verify the findings in this study .
